# Compression Properties of Interlayer and Intralayer Carbon/Glass Hybrid Composites

**DOI:** 10.3390/polym10040343

**Published:** 2018-03-21

**Authors:** Qingtao Wang, Weili Wu, Wei Li

**Affiliations:** 1 College of Textiles, Donghua University, No. 2999, Northern Renmin Rd, Songjiang District, Shanghai 201620, China; a19870628wqt@126.com (Q.W.); 1152003@mail.dhu.edu.cn (W.W.); 2 Key Lab of Textile Science & Technology, Ministry of Education, No. 2999, Northern Renmin Rd, Songjiang District, Shanghai 201620, China; 3 Center for Civil Aviation Composites, No. 2999, Northern Renmin Rd, Songjiang District, Shanghai 201620, China

**Keywords:** carbon/glass hybrid composites, compressive properties, interlayer hybrid, intralayer hybrid

## Abstract

The compression properties and mechanisms of interlayer and intralayer Carbon/Glass (C/G) hybrid composites were investigated in this work. As revealed from the experimental results, the compression modulus increases linearly with the increase of carbon fiber content, following the rule of mixtures (ROM). The C/G hybrid ratio is regarded as the decisive factor for the compression modulus of hybrid composites. The positive mixing effect exists on compression strength for interlayer and intralayer hybrid composites, whereas the experimental values are above the theoretical calculation values. The compressive strength of interlayer hybrid composites taking on various hybrid structures differs largely at the same mixed ratio, at which the compressive strength of glass fiber sandwiching carbon fiber is higher than that of carbon fiber sandwiching glass fiber. Through comparing interlayer and intralayer hybrid composites, the impact exerted by layer structures on the compressive strength of interlayer hybrid composites is higher than that of intralayer hybrid composites, which leads to more designable characteristics for interlayer hybrid composites. This work makes it possible to optimize the compression strength of interlayer hybrid structures so that it achieves or basically exceeds pure carbon fiber composites.

## 1. Introduction

Fiber-reinforced composites, by virtue of high specific strength, high modulus, and fatigue resistance, are widely applied in the aerospace, automotive wind power, etc. fields [1]. Carbon fiber composites take on high strength and modulus, whereas the fracture strain is small with insufficient impact resistance [2]. This defect could be a problem for the use of carbon-reinforced structures under compression or flexural loading. Besides this, the high cost of carbon fiber limits the widespread application of carbon fiber composites in the industry [3,4], which is the reason why carbon fiber is only popular in luxury products [5]. The modulus of glass fiber is lower relative to carbon fiber composites, but the breaking elongation is large, which leads to comparatively better impact resistance [6]. It is believed that a combination of carbon and glass fiber can make the best use of their advantages and make up for the deficiencies [7,8]. This type of material made from two or more reinforcements is called a hybrid composite. A mixture of Carbon/Class (C/G) can achieve the purpose of optimizing the fracture strain and modulus of composites [7,9]. So far, study on C/G hybrid composites has mainly focused on short fiber, sandwich, interlayer, and intralayer hybrids, where interlayer and intralayer hybrid structures are the main hybrid forms [10].

Research on the mechanical behaviors of hybrid composites primarily emphasizes the tensile and flexural properties of interlayer hybrid composites, and most of them are performed on various hybrid structures and mixed ratios under a single hybrid form [11,12,13,14,15,16,17,18,19,20,21,22]. The compression behavior of a composite is one of the most critical mechanical properties; however, there are few studies on the compressive properties of hybrid composites, which mainly research the compression behavior of interlayer hybrid composites [23,24]. S.F. Hwang [23] studied the compression properties of interlayer hybrid composites, and revealed that an increase in glass fiber content in hybrid composites can decrease the compressive strength. A similar conclusion can be confirmed by S. Chandra [25], who studied a C/G hybrid composite cylinder. S. Kedar [26] investigated the compression properties of a carbon and glass woven hybrid composite, and the result presented that a decrease in compressive strength of hybrid composites, compared with a carbon/epoxy composite, is not evident. M.H. Ikbal [27] found that the compressive strength of an intralayer hybrid structure is a bit better than that of an interlayer hybrid structure. M.T. Dehkordi [28] investigated the compressive strength of Basalt/Nylon interlayer hybrid composites, and revealed that hybrid structures have no effect on compressive strength; however, S.F. Hwang [24] found the compressive strength of the local symmetrical layer to be lower than that of the global symmetrical layer. Another study indicates that the compressive strain of sandwich hybrid specimens shows a 380% positive hybrid effect with regard to carbon fiber laminates [29].

Reports on the mechanical properties of intralayer C/G hybrid composites are limited; especially, little work has been done on the compression properties of intralayer hybrid. In our work, the interlayer and intralayer hybrid structures were designed systematically and the compression performances of the hybrid composites were investigated using experiments and compared with the values from theoretical calculations.

## 2. Materials and Methods

### 2.1. Experimental Materials

**Figure 1:**
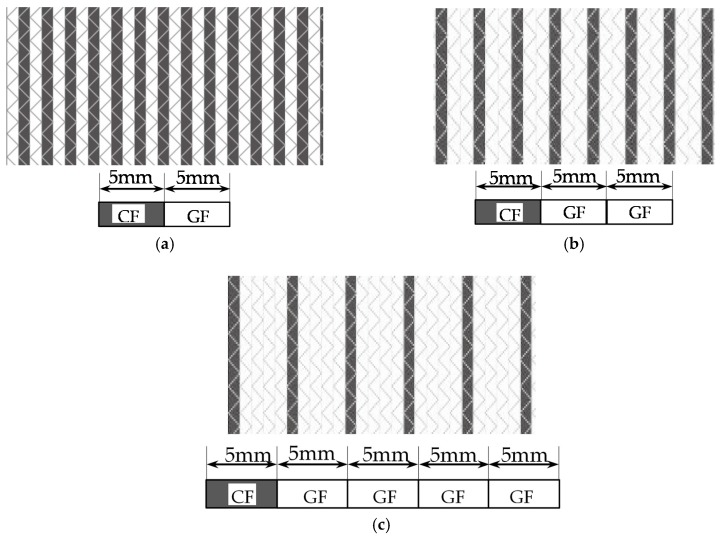
Schematic structures of three types of non-crimp fabrics (NCFs): (**a**) C–G; (**b**) C–G–G; (**c**) C–G–G–G–G.

T620SC-24K-50C carbon fiber supplied by TORAY Inc., ECT469L-2400 glass fiber from CPIC glass fiber Inc., and 2511-1A/BS epoxy resin from SWANCOR Inc. (Shanghai, China), were adopted in this work. Table 1 reports the mechanical parameters of the raw materials and specifications and structures of five unidirectional warp-knitted fabrics (Non-Crimp Fabric, NCF), including a pure carbon fiber fabric, a glass fiber fabric, and three kinds of hybrid fabrics with various C/G ratios, are presented in Table 2 and Figure 1, respectively.

### 2.2. Hybrid Scheme Design

#### 2.2.1. Interlayer Hybrid Composites

Interlayer hybrid structures consist of four C/G hybrid ratios as 1:1, 1:2, 1:3, and 1:4. Various hybrid structures are formed at the same ratio through altering the stacking configurations of carbon and glass fiber layers. Interlayer hybrid structures are presented in Table 3.

#### 2.2.2. Intralayer Hybrid Composites

Intralayer hybrid schemes consist of three hybrid fabrics with three mixed ratios. Four layers of a laminate were adopted to reduce the influence of the boundary effect. Various dispersion degrees were attained by the dislocation arrangements of hybrid fabrics at the same mixed ratio. Intralayer schemes are presented in Table 4.

### 2.3. Compression Tests

The vacuum-assisted resin transfer molding process (VARTM) was adopted to prepare composites, and fiber volume content was maintained at 50%. A compression test in the 0 and 90 degree directions was performed at speed of 1.3 mm/min according to the standard ASTM D6641. The force attenuation rate, considering the difference of speed and mode of carbon and glass fiber under failure, is established at 50% as the testing end parameter.

**Figure 2:**
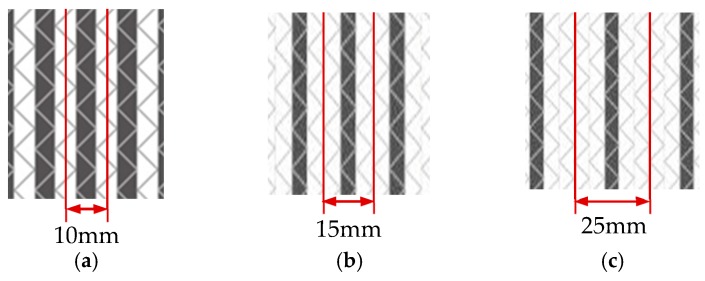
Schematics of sample cutting: (**a**) [C–G]; (**b**) [C–G–G]; (**c**) [C–G–G–G–G].

The specimen width of interlayer hybrid composites was set to 12 mm following ASTM D6641; the width of compression specimens was established at the width of a single cell of the hybrid structures. Carbon fiber and glass fiber should be symmetrically distributed to reduce the impact of asymmetric structures. Sample cutting diagrams are presented in Figure 2.

## 3. Results and Discussions

### 3.1. Compression Performances of Interlayer Hybrid Composites

**Figure 3:**
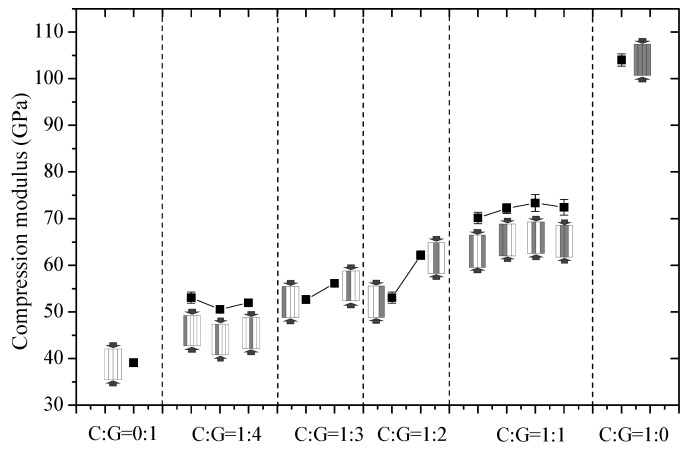
Compression modulus of different stacking sequences with various C/G ratios.

**Figure 4:**
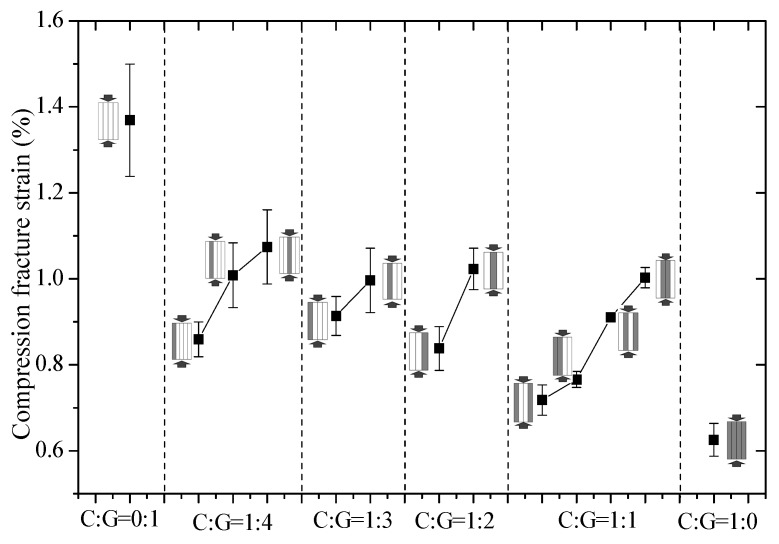
Compression fracture strain of different stacking sequences with various C/G ratios.

**Figure 5:**
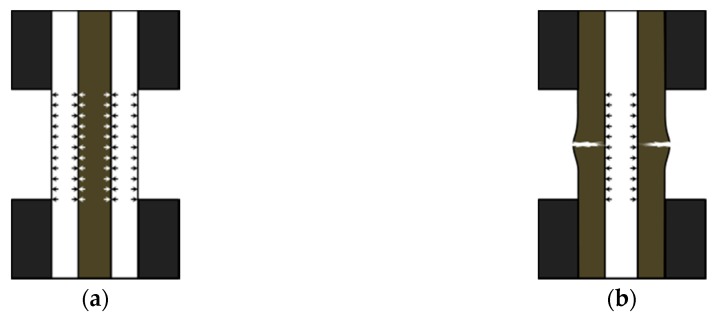
Internal stress diagram with (**a**) Carbon fiber inside, and (**b**) Carbon fiber outside under compression.

**Figure 6:**
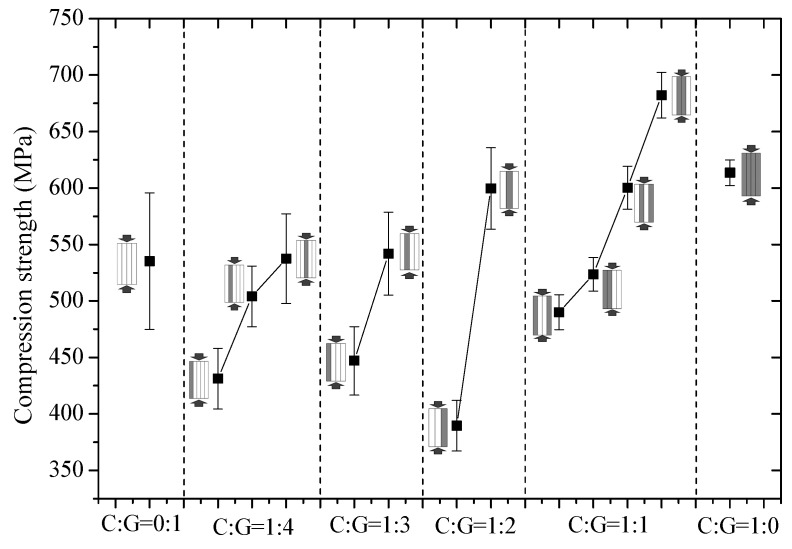
Compression strength of different stacking sequences with various C/G ratios.

The compression modulus, compressive fracture strain, compression mechanism and compressive strength of interlayer hybrid composites are presented in Figure 3, Figure 4, Figure 5 and Figure 6.

Figure 3 indicates the effect of mixed ratios and stacking sequences on the compressive modulus of interlayer hybrid composites. It was found that an increase in carbon fiber content improves the modulus of interlayer hybrid composites, whereas the impact exerted by various stacking sequences on the compression modulus is not evident at the same mixed ratio.

The results, presented in Figure 4, indicate that the compressive fracture strain of interlayer hybrid composites takes on an evident downward trend as carbon fiber content increases. The stacking sequence has a significant influence on the compressive strain at the same mixed ratio. As accordingly found, the compression fracture strain of an interlayer hybrid composite is comparatively large as glass fiber tends to be in the outer layer and carbon fiber in the inner layer, such as [G–C–C–G], [G–C–G], and [G–G–C–G–G]. Besides, compression fracture strain is small while the carbon fiber is all distributed in the outer layers, such as [C–G–G–C], [C–G–G–G–G], [C–G–G–G], and [C–G–G].

The internal stress states of interlayer hybrid structures under compression with carbon and glass fiber distributed in various layers are presented in Figure 5. Internal carbon fiber is subject to the inward extrusion force from external glass fiber with glass fiber in the outer layer and carbon fiber in the inner layer as presented in Figure 5a. Given this, it is difficult to deform the inner layer. The compressive fracture strain of glass fiber, additionally, is higher than that of carbon fiber, which continues to provide a certain amount of interlayer normal stress and prevents the collapse failure of the carbon layer. Figure 5b presents the structure with carbon fiber in the outer layer and with glass fiber in the inner layer, and the outside carbon layer provides a certain amount of interlayer normal stress to the inner glass fiber layer at the initial phase under compression. Yet the carbon fiber layers fail earlier and the glass fiber layers merely assume the compression force, which makes the strength comparatively low.

As observed in Figure 6 through referencing the foregoing failure mechanisms, there is no indication that the compressive strength of interlayer hybrid composites will increase linearly as carbon fiber content increases, whereas the stacking sequence of carbon and glass fiber greatly impacts compressive strength. Excellent compressive strength can be obtained by optimizing the mixed ratio and stacking sequences, inclusive of glass fiber distribution in the outer layer.

**Figure 7:**
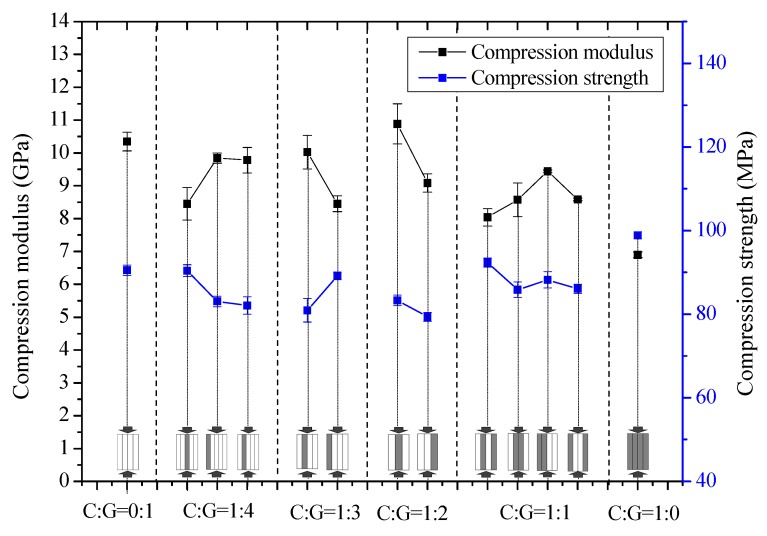
90-degree direction compression modulus and strength of different stacking sequences with various C/G ratios.

The results on the compression modulus and strength with various mixed ratios and hybrid structures in the 90-degree direction are presented in Figure 7. No evident impact is exerted by mixing ratios and the hybrid structure of C/G hybrid composites on the 90-degree compression modulus and strength. The compression properties of the 90-degree direction are primarily determined by the compression property of the resin given that the fiber is all arranged along the 0-degree direction.

### 3.2. Compression Performances of Intralayer Hybrid Composites

**Figure 8:**
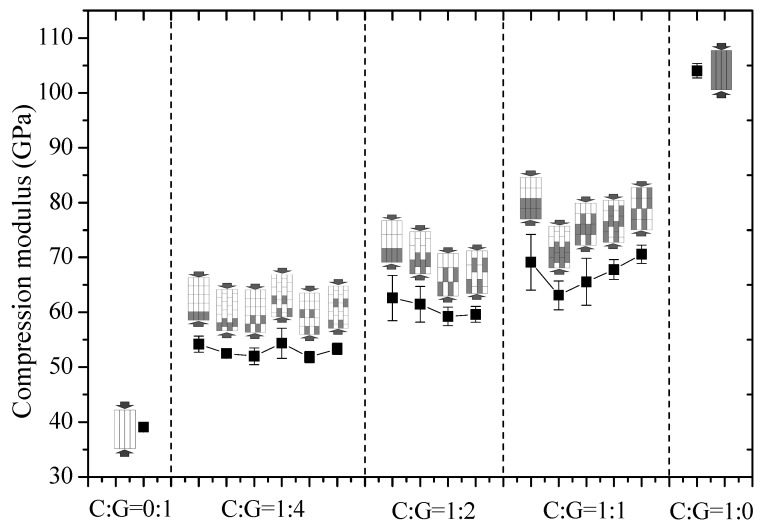
Compression modulus of different stacking sequences with various C/G ratios.

The compression modulus of intralayer hybrid composites with various mixing ratios and hybrid structures are presented in Figure 8. It was found that the modulus of intralayer hybrid composites increases progressively as the carbon fiber content increases. However, the impact of various layer structures on the modulus of a hybrid composite is not significant at the same C/G mixed ratio.

**Figure 9:**
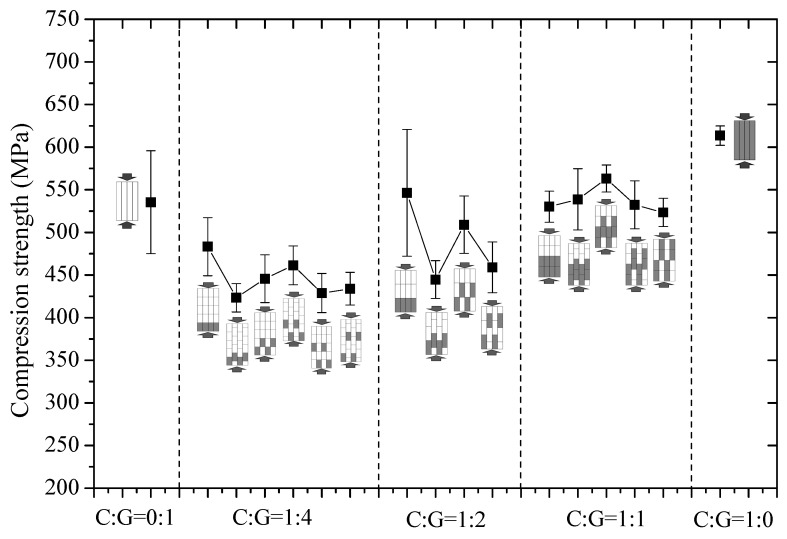
Compression strength comparison of different stacking sequences on various C/G ratios.

The compressive strength of hybrid composites with various mixing ratios and hybrid structures is presented in Figure 9. As indicated in the results, the compressive strength of interlayer hybrid composites is below that of both carbon and glass fiber composites. Moreover, alterations in carbon fiber content and hybrid structures at the same hybrid ratio exert no evident impact on the compressive strength.

### 3.3. Experimental and Theoretical Values of Compressive Strength and Modulus

Carbon fiber within hybrid composites fails firstly due to the low fracture strain, while the compressive loading exceeds the carbon fiber’s maximum strain, and glass fiber still assumes the residual load until it is damaged. Formulas for calculating stress of hybrid composites before and after the fracture of carbon fiber are presented as (1) and (2) [30] following the rule of mixture (ROM).

(1) $$\text{Before carbon fiber fracture}:\;\sigma_{\mathrm{HY}}=\left(V_{C}E_{C}+V_{G}E_{G}\right)\varepsilon$$

(2) $$\text{After carbon fiber fracture}:\;\sigma_{\mathrm{HY}}=V_{G}E_{G}\varepsilon$$

where $\sigma_{\mathrm{HY}}$ denotes the compression stress of a hybrid composite (MPa), $V_{C}$, $V_{G}$ represent the volume content of carbon fiber and glass fiber composite (%), respectively, $E_{C}$, $E_{G}$ refer to the compression modulus of carbon fiber and glass fiber composite (GPa), respectively, and ε represents the strain of the hybrid composite (%).

**Figure 10:**
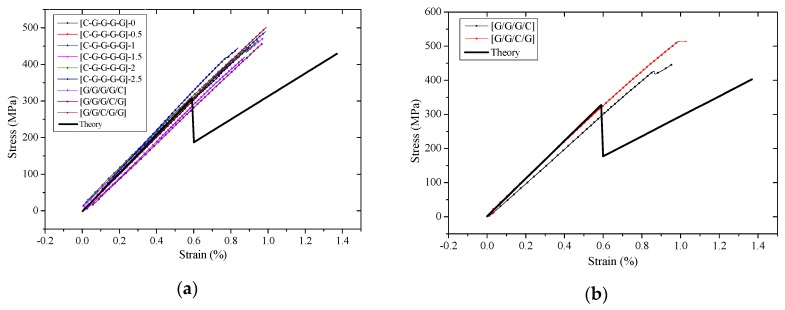

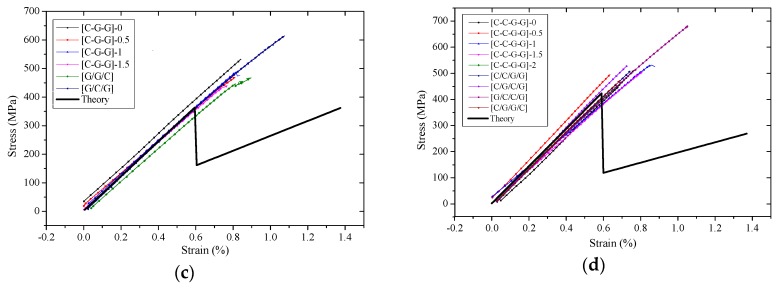
Experimental and theoretical compression stress-strain curves of interlayer and intralayer hybrid composites at various hybrid ratios: (**a**) C:G=1:4; (**b**) C:G=1:3; (**c**) C:G=1:2; (**d**) C:G=1:1.

Comparisons of compressive stress-strain curves from experimental and theoretical results are shown in Figure 10. The experimental modulus is indicated to be complying well with the theoretical value in the initial phase. Yet as a higher loading is applied, the experimental compressive strength appears to differ apparently from the theoretical curves and present a ladder-type change. Compressive collapse leads to a sample’s failure and the failure process is comparatively slow. After crushing failure, carbon composites with low fracture strain, unlike the rapid failure in the tensile process, still assume a certain force until the sample completely fails.

The mechanical properties of hybrid composites present various hybrid effects, while some structures can enhance their mechanical properties, and some structures may weaken their properties. Accordingly, this work here introduces the ROM to evaluate the hybrid effect. As indicated by the ROM, the mechanical property of a hybrid structure is calculated following the mixed ratio of two materials [19]. The calculation formula for the compression modulus of hybrid composites is presented below:

(3) $$E_{ROM}=V_{C}E_{C}+V_{G}E_{G}$$

where $E_{ROM}$ denotes the compression modulus of hybrid composites (GPa).

**Figure 11:**
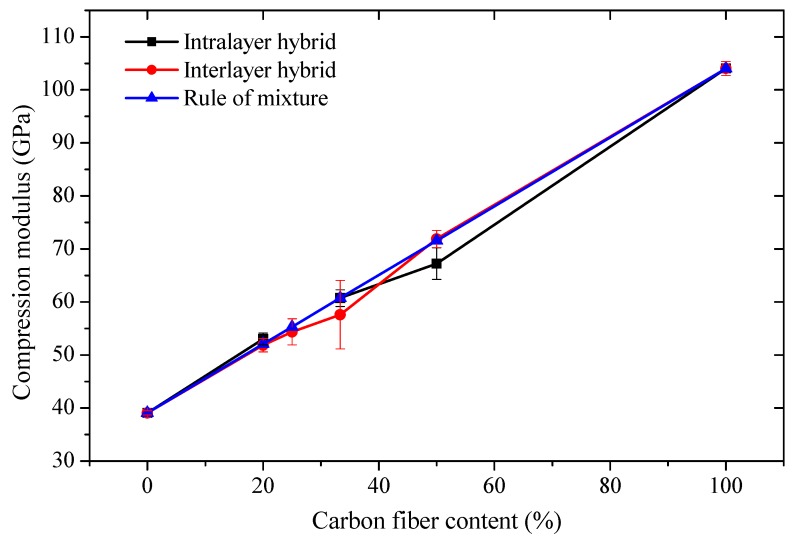
Interlayer and intralayer compression modulus attained by experiment and rule of mixture (ROM).

Figure 11 presents the comparison between the experimental compression modulus and the theoretical values. It was found that the theoretical values comply well with the experimental values.

**Figure 12:**
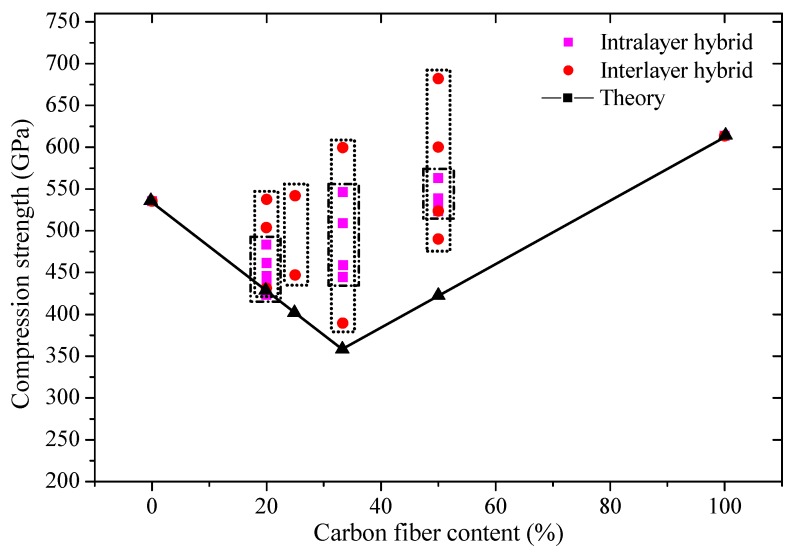
Interlayer and intralayer compression strength obtained by experiment and ROM.

The experimental and theoretical compression strength of hybrid composites is presented in Figure 12. In theory, the compression strength tends to decrease first and then increase as the content of carbon fiber increases. As indicated in the theoretical analysis, the compression strength of a hybrid structure is minimized for the 1:2 C/G mixed ratio. The experimental compression strength of the interlayer and intralayer hybrid composites is above the theoretical values, which demonstrates a positive hybrid effect, and the compression strength takes on an increasing trend as the carbon fiber content increases. The impact exerted by variations in layer structures of an interlayer hybrid on the compression strength is greater than an intralayer hybrid at the same mixed ratio. The compression strength of the interlayer structure, such as [G–C–C–G] and [G–C–G], is deemed to be basically stronger than or equal to pure carbon fiber composites which makes it possible to achieve higher compressive strength with less carbon fiber content.

## 4. Conclusions

Compressive properties of hybrid composites were investigated in this work, and failure mechanisms were analyzed.

The following conclusions were made:

- The compression modulus of interlayer and intralayer hybrid composites is determined by the mixed ratio.
- Alterations in layer structures merely impact the compressive strength of the interlayer hybrid composites, which mainly manifest as compression strength, fracture strain and strength of an interlayer hybrid composite with glass fiber sandwiching carbon fiber above that of carbon fiber sandwiching glass fiber.
- There is no evident impact of mixing ratios and hybrid structure of C/G interlayer hybrid composites on compression modulus and strength under the 90-degree compression loading.
- As indicated through comparing experimental results and theoretical calculation values for interlayer and intralayer hybrid composites, the experimental compressive modulus is consistent with theoretical values calculated via the ROM, while the experimental compressive strength surmounts the theoretical values and exhibits a positive hybrid effect.
- Moreover, interlayer hybrid composites provide more excellent mechanical properties than intralayer hybrid structures, which make it possible for interlayer hybrid composites to attain higher strength on the premise of using less carbon fiber.

## Figures and Tables

**Table 1:** Constituent materials and selected properties.

Material	Tensile Strength (MPa)	Tensile Modulus (GPa)
CPIC ECT469L-2400 Glass Fiber	2366	78.7
TORAY T620SC-24K-50C Carbon Fiber	4175	234
SWANCOR 2511-1A/BS Epoxy Resin	73.5	3.1

**Table 2:** Specifications for hybrid fabrics.

Fabric Type	Areal Density (g/m^2^)		Ratio of Carnon/Glass (C/G)
	Carbon Fiber	Glass Fiber	
Carbon	728.3	0	1:0
Glass	0	944.9	0:1
C–G	364.2	472.4	1:1
C–G–G	242.8	629.9	1:2
C–G–G–G–G	145.7	755.9	1:4

**Table 3:** Stacking configurations of interlayer hybrid structures.

C/G Hybrid Ratios	Stacking Sequences			
C:G=1:1	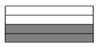	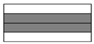	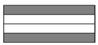	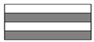
	[G/G/C/C]	[G/C/C/G]	[C/G/G/C]	[G/C/G/C]
C:G=1:2	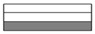	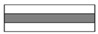		
	[G/G/C]	[G/C/G]		
C:G=1:3	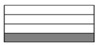	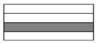		
	[G/G/G/C]	[G/G/C/G]		
C:G=1:4	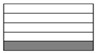	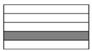	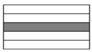	
	[G/G/G/G/C]	[G/G/G/C/G]	[G/G/C/G/G]	

**Table 4:** Stacking configurations of intralayer hybrid structures.

Hybrid Fabric	Stacking Sequences			
C–C–G–G C:G=1:1	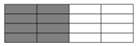	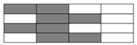		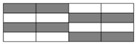
	[C–C–G–G]-0	[C–C–G–G]-1		[C–C–G–G]-2
	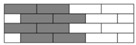	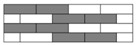		
	[C–C–G–G]-0.5	[C–C–G–G]-1.5		
C–G–G C:G=1:2	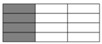	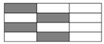	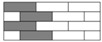	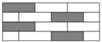
	[C–G–G]-0	[C–G–G]-1	[C–G–G]-0.5	[C–G–G]-1.5
C–G–G–G–G C:G=1:4	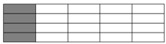	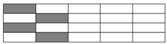		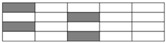
	[C–G–G–G–G]-0	[C–G–G–G–G]-1		[C–G–G–G–G]-2
	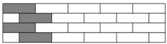	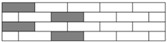		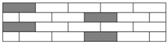
	[C–G–G–G–G]-0.5	[C–G–G–G–G]-1.5		[C–G–G–G–G]-2.5
